# Alzheimer’s Disease patient stratification using iPSC‐derived hindbrain organoids as a novel pre‐clinical tool for drug testing

**DOI:** 10.1002/alz.089065

**Published:** 2025-01-03

**Authors:** Cristina Zivko, Ram Sagar, Ariadni Xydia, Constantine G. Lyketsos, Vasiliki Machairaki

**Affiliations:** ^1^ Johns Hopkins University School of Medicine, Baltimore, MD USA; ^2^ Johns Hopkins Bayview Medical Center, Baltimore, MD USA; ^3^ School of Medicine, Johns Hopkins University, and Johns Hopkins Bayview Medical Center, Baltimore, MD USA; ^4^ Johns Hopkins University, Baltimore, MD USA

## Abstract

**Background:**

By 2050 the number of Alzheimer’s Disease (AD) patients is projected to exceed 150 million worldwide. AD is an incurable, insufficiently understood, and devastating neurodegenerative disease, with high patient heterogeneity in terms of progression, clinical manifestation (including neuropsychiatric symptoms, NPS) and, importantly, responsiveness to treatment options.[1] In the last 20 years, 98% of clinical trials for AD have failed, highlighting the urgent need to drastically change pre‐clinical research to develop better predictors of drug safety and effectiveness.[2]

This is where our precision medicine approach of organoid in vitro models can be a game changer.

**Method:**

Human peripheral blood mononuclear cells from healthy volunteers (n = 6) and AD patient with (n = 12) and without NPS (n = 12) were reprogrammed into induced pluripotent stem cells (iPSCs)[3] and subsequently differentiated into hindbrain organoids, containing serotonin (5‐HT) producing neurons.

**Result:**

Extensive characterization and validation experiments were conducted to assess the quality of the iPSCs and of the 5‐HT‐organoids. These include karyotyping, fluorescent microscopy and flow cytometry to reveal the presence of pluripotency (OCT4, NANOG, TRA‐1‐60) and neuronal markers (TUJ1, TPH2, 5‐HT), real time qPCR, ELISA measurement of 5‐HT levels, size and circularity over 6 weeks of differentiation, for which we developed an in‐house algorithm in Python. The 5‐HT‐organoids were treated with a selective serotonin reuptake inhibitor acutely (1 h) and for 1 week, upon which we could stratify the 30 individuals according to drug responsiveness. Shotgun proteomics analysis was performed on the treated and non‐treated 5‐HT‐organoids from all 30 individuals by LC/MS to reveal distinctive relative abundance profiles. Experiments were run in biologically independent triplicates for 6 cell lines (3 healthy, 3 AD). The results from that training set were used to classify analytical results from the other 24 lines for AD biomarker discovery, as well as for drug responsiveness and effect.

**Conclusion:**

Highly reproducible, people‐specific 5‐HT‐organoids were thus used to study inter‐patient phenotypical variability and drug response diversity in a powerful, novel pre‐clinical tool.

**References**:

[1] Kim CK et al (2022). J Alzheimers Dis. 2022;87(1):83‐100. https://doi.org/10.3233/JAD‐215699.

[2] Lyketsos CG et al (2011). J. Alzheimers Assoc. 7(5):532‐539. https://doi.org/10.1016/j.jalz.2011.05.2410

[3] Sagar R et al (2023). Cells. 12(15):1990. https://doi.org/10.3390/cells12151990.

